# lncRNA SNHG4 enhanced gastric cancer progression by modulating miR-409-3p/CREB1 axis

**DOI:** 10.32604/or.2024.042281

**Published:** 2024-12-20

**Authors:** ZHOUYANG CHENG, YUCHEN HUA, YANG CAO, JUN QIN

**Affiliations:** 1 Department of General Surgery, Affiliated Hospital of Nantong University, Nantong, 226001, China; 2 Department of Surgery, Affiliated Hospital of Nantong University, Medical School of Nantong University, Nantong, 226001, China; 3 Department of Operation, Affiliated Hospital of Nantong University, Nantong, 226001, China

**Keywords:** Gastric cancer, Small nucleolar RNA host gene 4 (SNHG4), MicroRNA-409-3p (miR-409-3p), cAMP responsive element binding protein 1 (CREB1)

## Abstract

**Objective:**

Gastric cancer (GC) is a globally common cancer characterized by high incidence and mortality worldwide. Advances in the molecular understanding of GC provide promising targets for GC diagnosis and therapy. Long non-coding RNAs (lncRNAs) and their downstream regulators are regarded to be implicated in the progression of multiple types of malignancies. Studies have shown that the lncRNA small nucleolar RNA host gene 4 (SNHG4) serves as a tumor promoter in various malignancies, while its function in GC has yet to be characterized. Therefore, our study aimed to explore the role and underlying mechanism of SNHG4 in GC.

**Methods:**

We used qRT-PCR to analyze SNHG4 expression in GC tissues and cells. Kaplan-Meier analysis was used to assess the correlation between SNHG4 expression and the survival rate of GC patients. Cellular function experiments such as CCK-8, BrdU, colony formation, flow cytometry analysis, and transwell were performed to explore the effects of SNHG4 on GC cell proliferation, apoptosis, cell cycle, migration, and invasion. We also established xenograft mouse models to explore the effect of SNHG4 on GC tumor growth. Mechanically, dual luciferase reporter assay was used to verify the interaction between SNHG4 and miR-409-3p and between miR-409-3p and cAMP responsive element binding protein 1 (CREB1).

**Results:**

The results indicated that SNHG4 was overexpressed in GC tissues and cell lines, and was linked with poor survival rate of GC patients. SNHG4 promoted GC cell proliferation, migration, and invasion while inhibiting cell apoptosis and cell cycle arrest *in vitro*. The *in vivo* experiment indicated that SNHG4 facilitated GC tumor growth. Furthermore, SNHG4 was demonstrated to bind to miR-409-3p. Moreover, CREB1 was directly targeted by miR-409-3p. Rescue assays demonstrated that miR-409-3p deficiency reversed the suppressive impact of SNHG4 knockdown on GC cell malignancy. Additionally, miR-409-3p was also revealed to inhibit GC cell proliferation, migration, and invasion by targeting CREB1.

**Conclusion:**

In conclusion, we verified that the SNHG4 promoted GC growth and metastasis by binding to miR-409-3p to upregulate CREB1, which may deepen the understanding of the underlying mechanism in GC development.

## Introduction

As a common malignant tumor, gastric cancer (GC) is the third most fatal tumor globally. GC is particularly prevalent in East Asia [1]. In fact, approximately 50% of the world’s GC patients are located in East Asia, and the incidence rate of GC in China is about six-fold higher relative to the United States. In China, the incidence of GC is 47 patients per 100000 people, which results in a huge economic and public health burden [2]. Most patients with early GC have no obvious symptoms, and a few people have nausea, vomiting, or upper gastrointestinal symptoms similar to ulcer disease, which is difficult to attract sufficient attention. With the growth of the tumor, more obvious symptoms appeared when the gastric function was affected, but they lacked specificity. Pain and weight loss are the most common clinical symptoms of advanced GC [3]. The main Current treatments for GC include surgery, radiotherapy, chemotherapy, and immune cell therapy [4]. The high mortality rate is largely due to poor access to early diagnosis and lack of effective intervention methods. The pathogenesis of gastric cancer is complex, including genetic factors, environmental factors and additional factors. Numerous studies have suggested that the occurrence of GC is associated with abnormal transcription [5]. This abnormality is not only limited to aberrant levels of protein-coding RNAs (mRNAs), but may also lead to the abnormal regulatory ability of genomic non-coding RNAs (ncRNAs), such as long non-coding RNAs (lncRNAs) [6], pseudogenes [7], and microRNAs (miRNAs) [8]. Therefore, the study of the pathogenesis of GC, especially the diagnosis and prognostic markers, has been paid increasing attention by researchers [9].

LncRNAs refer to a form of non-coding RNAs over 200 nucleotides in length, and participate in diverse important regulatory process such as chromatin modification and transcriptional activation. LncRNAs have been proved to participate in various tumor-related processes [10–12]. MiRNAs refer to small non-coding transcripts with an average length of only 22 nucleotides. There are more than 1000 miRNAs expressed in humans, and 60% of genes in the human body can be regulated by miRNAs. Numerous studies have validated [13,14] that miRNAs affect the malignant behaviors of tumor cells. Hence, dyregulation of diverse miRNAs is observed in many tumor diseases, including GC. MiRNAs can degrade their target genes or inhibit the translation of target genes by binding to the 3’-untranslated regions (3’-UTRs) of the target genes [15]. As a potential marker for early diagnosis or prognosis of tumors, miRNAs can help design novel anti-cancer strategies [16]. Some lncRNAs can competitively bind to miRNAs as competitive endogenous RNAs (ceRNAs) and play a key post-transcriptional regulatory role in regulating tumor progression [17–19].

Previous studies have investigated the role of lncRNAs in GC, and found a close connection between lncRNAs and miRNAs. For example, Ding et al. [20] discovered that the lncRNA TP73-AS1 can help accelerate the invasion of GC by modulating the miR-1945p/SDAD1 axis. Besides, lncRNAs have also been confirmed to be associated with the clinical prognosis of GC patients, such as Zhou et al. [21] identified low expression of the lncRNA LET is an independent risk factor for poor prognosis in GC patients. Small nucleolar RNA host gene 4 (SNHG4) has been a research hotspot in tumor-related diseases, especially in prostate cancer [22], lung cancer [23], and human osteosarcoma [24]. Up till now, there is little published data on the relationship between the lncRNA SNHG4 and GC.

cAMP-response element binding protein (CREB1) is located on human chromosome 2q34 and has been certified to be an oncogene in a variety of cancers [25]. Abnormal CREB1 expression is linked to the occurrence and development of tumors by modulating the expression of downstream genes including apoptosis-related genes, cycle-related genes, invasion-related genes, as well as signal transduction proteins [26]. For GC, CREB1 expression has been reported to be up-regulated and is associated with distant metastasis as well as poor outcome in patients with GC, suggesting the oncogenic potential of CREB1 in GC [27,28].

Herein, we attempted to examine the expression of the lncRNA SNHG4 in GC, and further explored the biological function and underlying mechanism of SNHG4 in GC cells. Our results demonstrate that SNHG4 modulates the miR-409-3p/CREB1 axis to exert its function in GC progression, indicating that the SNHG4/miR-409-3p/CREB1 network can be a promising target for GC therapy.

## Materials and Methods

### Bioinformatics prediction

RNA-seq data were obtained from The Cancer Genome Atlas (TCGA) database (https://www.cancer.gov/ccg/research/genome-sequencing/tcga). Data were processed using the limma package R software was implemented to explore the differentially expressed genes in GC samples compared with normal tissues. *p* value less than 0.05 and log_2_(FC) >1 or <−1 were regarded as statistical significance. Next, the differentiated expression of SNHG4 was visualized by “ggpubr” package.

### Patient tissues and cell culture

We collected 60 pairs of GC patients that underwent surgery in our hospital with complete data records. All subjects signed an informed consent form. The Ethics Committee of our hospital authorized the present study. Human gastric mucosal epithelial cells (GES-1) and gastric cancer cells (SGC-7901, AGS, MKN28, BGC-823, MGC803, and HGC-27) were offered by the Shanghai Institute of Cellular Cells at the Chinese Academy of Sciences (Shanghai, China). Human GC cell lines and human gastric mucosal epithelial cells were routinely cultured in RPMI-1640 medium (Sigma-Aldrich, St Louis, MO, USA) containing 10% fetal bovine serum (Thermo Fisher, Rockford, IL, USA) at 37°C and 5% CO_2_ atmosphere. Prior to the test, all cells were identified by STR, and no cells had received mycoplasma infection.

### Plasmids transfection

The transfection was carried out after GC cells (SGC-7901 and AGS) reaching the logarithmic growth phase. After digestion with trypsin (Sigma-Aldrich, St Louis, MO, USA), GC cells were suspended in 1640 medium to 1 × 10^5^ cells/mL, and plated onto six-well plates. Next, cells were then cultured at 37°C in a 5% CO_2_ incubator for 24 h. Si-SNHG4 or SNHG4 ov (GenePharma, Shanghai, China) was transfected into the SGC-7901 cell line or AGS cell line, respectively. Negative control of small RNA and ov was utilized as vehicle. MiR-409-3p mimics/inhibitor and CREB1 ov (GenePharma, Shanghai, China) were transfected in the SGC-7901 cell line. The transfection method was referred to as the Lipofectamine 2000 transfection reagent (Thermo Fisher, Rockford, IL, USA) manual. After 48 h, the transfection effect was analyzed.

### Quantitative reverse transcription polymerase chain reaction (qRT-PCR)

The clinical fresh samples and GC cancer cells were gathered 48 h post-transfection. Additionally, TRizol reagent (Thermo Fisher, Rockford, IL, USA) was applied for total RNA extraction from tumor samples as well as cultured cells. Then cDNA was synthesized using SuperScript II (Vazyme, Nanjing, China). Next, the RNA expression of the SNHG4 as well as CREB1 was analyzed with the SYBR Green assays on the ABI 7900HT RealTime PCR System (Thermo Fisher, Rockford, IL, USA) with their expression normalized to GAPDH. MiR-409-3p levels were quantified utilizing TaqMan MicroRNA Assays (Thermo Fisher, Rockford, IL, USA) and normalized to U6. The 2^−ΔΔCt^ method was applied to evaluate the levels of RNAs, and primer sequences were provided in Table 1, and this experiment was repeated thrice.

**Table 1:** Primers used in this study

Name	Sequences
SNHG4 forward primer	5′-GCAGGTGACAGTCTGCATGT-3′
SNHG4 reverse primer	5′-TTTTAAGTCCCCTACCCCCATC-3′
CREB1 forward primer	5′-CCAGCAGAGTGGAGATGCAG-3′
CREB1 reverse primer	5′-GTTACGGTGGGAGCAGATGAT-3′
GAPDH forward primer	5′-GACTCATGACCACAGTCCATGC-A′
GAPDH reverse primer	5′- AGAGGCAGGGATGATGTTCTG-3′

### Western blot

Levels of CREB1, epithelial-mesenchymal transition (EMT)-related proteins, the cyclin pathway-related protein, and the apoptosis-related proteins in GC cells were subject to western blot. Total protein in each group was extracted using radioimmuno-precipitation assay (RIPA) protein lysis buffer (Thermo Fisher, Rockford, IL, USA) and protein concentration was detected through the bicinchoninic acid (BCA) kit (Sigma-Aldrich, St Louis, MO, USA). After electrophoretic separation of target protein through 10% Sodium dodecyl sulfate-polyacrylamide gel electrophoresis (SDS-PAGE) gel, samples were blotted on polyvinylidene fluoride (PVDF, Millipore, Billerica, USA) membranes. The corresponding primary antibody CREB1 (12208-1-AP; 1:2000; Proteintech; Chicago; USA), Vimentin (ab92547; 1:2000; Abcam; Cambridge; UK), E-cadherin (ab181296; 1:1000, Abcam; Cambridge; UK), Cyclin D1 (ab16663; 1:200; Abcam; Cambridge; UK), Bax (ab32503; 1:1000; Abcam; Cambridge; UK), Bcl-2 (ab32124; 1:1000; Abcam; Cambridge; UK) with GAPDH (ab181602; 1:10000; Abcam; Cambridge; UK) as a loading control were added to co-incubate with the membrane for 12 h at 4°C. After phosphate buffer saline (PBS, Sigma-Aldrich, St. Louis, MO, USA) washing, the membranes were cultured with the secondary antibody at 37°C for 60 min. Finally, the target band was scanned and visualized on a gel imager. The experiment was repeated 3 times.

### Cell counting kit-8 (CCK-8) assay

For CCK-8, GC cells post indicated transfected were reaped and inoculated onto 96-well plates (100000 cells per well). Next, 100 μL medium was added per well. Then, the culture plate was incubated for 24, 48, 72, and 96 h, and each well was supplemented with CCK-8 solution (10 μL, Biosharp, Beijing, China) and maintained for 2 h before detection. The OD 450 nm values were detected using an enzyme labeling instrument to calculate cell viability. This experiment was repeated 3 times.

### Bromodeoxyuridine (BrdU) assay

Briefly, transfected GC cells were inoculated into a 96-well plate (10000 cells per well). Then each well was supplemented with 15 μL of BrdU solution (Thermo Fisher, Rockford, IL, USA) and cultured for 2 h at 37°C. After replacing the supernatant with denaturated solution (100 μL) and incubation for 10 min, the mixture was added with the BrdU antibody, corresponding secondary antibody and tetramethylbenzidine and maintained for 30 min. OD value at 370 nm was detected using a microplate reader (Thermo Fisher, Rockford, IL, USA). This experiment was independently conducted 3 times.

### Transwell assay

For cell invasiveness assessment, the top well of Boyden chamber was pre-coated with diluted matrigel (200 μL) and dried overnight. Then, the single cell suspension of transfected GC cells was prepared. The top chamber was then added with the transfected cells with serum-free cultured medium, and medium containing 10% FBS was supplemented into bottom chamber. After culturing the cells at 37°C for 1 day, the chambers were rinsed by PBS thrice. Cells were processed with 95% ethanol, rinsed by PBS, and subjected to crystal violet staining solution (Beyotime, Shanghai, China) for 10 min. A microscope was applied to capture the images and the average cell number was calculated. For the evaluation of GC cell migration ability, the top chamber was not pre-coated with matrigel, and the other steps were the same as mentioned above. This experiment was repeated 3 times.

### Cell proliferation assessment

GC cells after transfection were reaped and grown into a 6-well plate (5000 cells per well). After incubation for 14 d with 5% CO_2_ at 37°C, the colonies were fixed using 4% paraformaldehyde (Sigma-Aldrich, St. Louis, MO, USA) and dyed using crystal violet (0.1%, Beyotime, Shanghai, China) for 30 min. Then, the light microscope was utilized for the calculation of the number of colonies. The experiment was conducted in triplicate.

### Apoptosis and cell cycle measurement

Flow cytometry analyses were conducted to determine levels of GC cell apoptosis. After digestion with trypsin followed by PBS rinsing twice, cells were re-suspended with 150 μL binding buffer. Then, annexin fluorescein isothiocyanate (FITC, 5 μL, Sigma-Aldrich, St. Louis, MO, USA) and propidium iodide (PI) dye solution (10 μL, Sigma-Aldrich, St. Louis, MO, USA) were added and maintained for 15 min and then analyzed by a flow cytometer. For the detection of cell cycle distribution, GC cells were transferred to 12-well plates, cultured for 24 h and fastened using ethanol (Sigma-Aldrich, St. Louis, MO, USA). Then cells were maintained for 7 h at 4°C, followed by PBS washing, and then subjected to PI staining for 30 min in the dark. Flow cytometry (Thermo Fisher, Rockford, IL, USA) was applied for cell cycle distribution analysis. This experiment was conducted in triplicate.

### Dual luciferase reporter assays

The binding sequences of SNHG4 or CREB1 with miR-409-3p were PCR-amplified, respectively. Wild-type (wt) SNHG4 or CREB1 were generated by inserting the amplified products into the pmirGLO vector (Promega, Madison, USA). Mutant (mut) SNHG4 and CREB1 were produced via site-directed mutagenesis of the binding sequences. Then SGC-7901 cells received the transfection of recombinant plasmids with miR-409-3p/miR-NC mimics. Cells were collected 48 h post transfection. Luciferase detection was carried out using a dual luciferase assay kit in accordance with the producer’s instructions.

### Tumor-bearing mouse models

Male BALB/C nude mice (5-weeks old, body weight 18 ± 2 g) were allowed to acclimatize to 22–24°C routinely. SGC-7901 and AGS cells (5 × 10^5^) were infected with either si-SNHG4 or SNHG4 ov, and mice received subcutaneous injection of these cells at the right side, respectively. The volume of mouse tumors was monitored every three d via the formula volume (mm^3^) = length (mm) × width (mm) × width (mm)/2. Mice were killed after 21 days post injection, and tumors were obtained. The Animal Ethics Committee of our hospital approved the animal study.

### Statistical analysis

The GraphPad v7 as well as SPSS 19.0 software was utilized for result analyses. The measurement data were presented as the mean ± standard deviation (SD). Categorical data were examined by chi-square test. Statistical difference of two groups or among three or more groups was analyzed with *t*-test or one-way analysis of variance (ANOVA), respectively. The survival outcome of GC patients was analyzed by Kaplan-Meier Survival analysis using log-rank test. *p* value less than 0.05 indicated statistical significance.

## Results

### LncRNA SNHG4 expression in GC samples and cells

**Figure 1:**
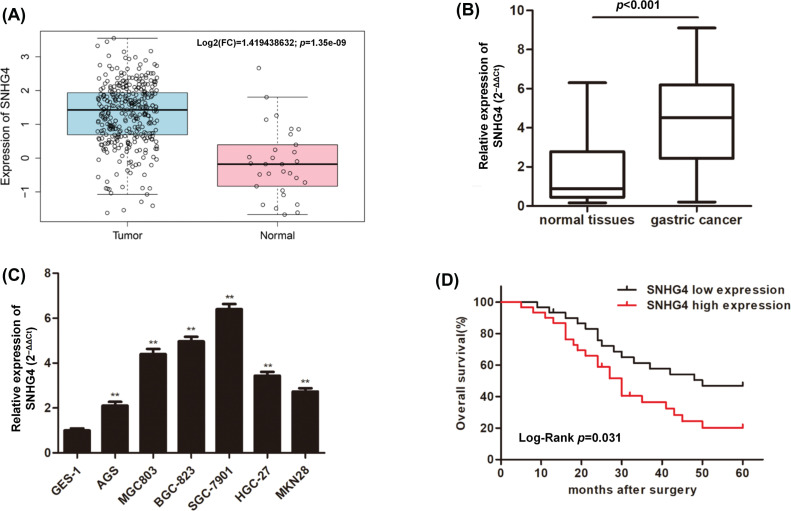
Upregulation of lncRNA SNHG4 in GC tissue samples and cell lines. (A) TCGA database shows SNHG4 expression pattern in GC. (B and C) SNHG4 expression in GC patient samples (n = 60) and cells measured by qRT-PCR. (D) Overall survival rate in GC patients with variable SNHG4 levels with Kaplan-Meier analyses. **p < 0.01.

To discover the role of SNHG4 in GC, we downloaded and analyzed the TCGA RNA-seq data, which indicated that SNHG4 was expressed at a higher level in GC tumor compared to adjacent normal tissues (Fig. 1A). Next, qRT-PCR determined the SNHG4 expression in GC samples (n = 60) as well as normal samples (n = 60) that we initially collected. The results were consistent with TCGA prediction (Fig. 1B). Furthermore, the SNHG4 was revealed to be expressed at higher levels in GC cells compared to GES-1 (Fig. 1C). Therein, SGC-7901 cell line demonstrated the highest SNHG4 levels, while AGS cell line showed the relative low SNHG4 levels across these six GC cell lines. Kaplan-Meier analysis of the relationship between high or low SNHG4 expression and the overall survival revealed that increased SNHG4 levels were associated with poorer GC survival rate (Fig. 1D). Additionally, we evaluated the whether SNHG4 levels were associated with clinico-pathologic characteristics of patients with GC. SNHG4 expression exhibited significant correlation with the lymph node metastasis (*p* = 0.008), tumor size (*p* = 0.004), as well as TNM stages (*p* = 0.002) (Table 2), with no significant relation to age, sex as well as histological grades.

**Table 2:** Correlation of SNHG4 expression with clinicopathological characteristics in GC patients

Characteristic	Case	SNHG4 expression		*p* value
		Low	High	
All case	60	30	30	
Age (years)				0.435
<60	23	10	13	
≥60	37	20	17	
Gender				0.432
Female	25	14	11	
Male	35	16	19	
Tumor size (cm)				***0.004***
<3 cm	27	19	8	
≥3 cm	33	11	22	
Histological grade				0.121
High	30	18	12	
Low-middle	30	12	18	
Lymph node metastasis				***0.008***
Negative	36	23	13	
Positive	24	7	17	
TNM stage				***0.002***
I–II	24	18	6	
III–IV	36	12	24	

### Silenced lncRNA SNHG4 inhibited the proliferation, migration, invasion and EMT and increased the apoptosis of GC cells

**Figure 2:**
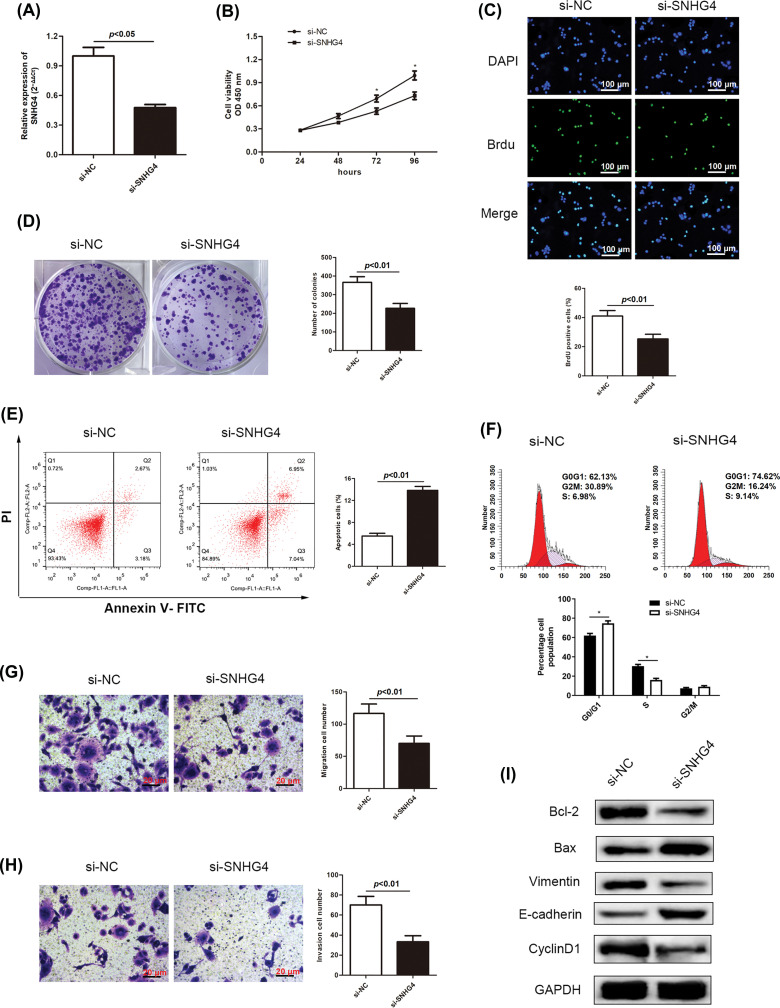
SNHG4 deficiency inhibits GC cell growth and metastasis. (A) SNHG4 knockdown in the SGC-7901 cell line. (B–D) Growth curves based on CCK-8, BrdU, and colony formation assays of SGC-7901 cell line transfected with si-SNHG4. (E) Cell apoptosis evaluated by flow cytometry. (F) Cell cycle distribution evaluated by flow cytometry. (G and H) Transwell assays examined invasion and migration ability of GC cells with absence of SNHG4. (I) Western blot measured proteins expression related to proliferation, EMT and apoptosis pathways. *p < 0.05.

Based on our previous finding that increased SNHG4 levels were associated with GC tumors, we constructed two cell lines transfected with si-SNHG4 in order to explore the effects of SNHG4 silence on the malignant behaviors of GC cells. Results from qRT-PCR assay confirmed that knockdown SNHG4 induced a down-regulation of SNHG4 in SGC-7901 cell line (Fig. 2A). From Figs. 2B–2D, we could see that SNHG4 knockdown induced a dramatic decrease in GC cell viability. Flow cytometry analysis was carried out to assess cell apoptosis and cell cycle distribution. Our results demonstrated that the apoptosis rate was markedly increased when SNHG4 was knockdown (Fig. 2E). Furthermore, we revealed that knockdown of SNHG4 increased cell cycle distribution in the G0/G1 phase (Fig. 2F). In addition, the effects of depleted SNHG4 expression on cell migration and invasion were also evaluated using transwell experiments. Reduced GC cell migration and invasion were observed in the SNHG4 knockdown group (Figs. 2G and 2H), According to the western blot, knockdown of SNHG4 inhibited Cyclin D1, Bcl-2 and Vimentin protein levels, enhanced Bax and E-cadherin protein levels (Fig. 2I).

### Overexpressed lncRNA SNHG4 accelerated the proliferation, migration, invasion and EMT and repressed the apoptosis of GC cells

**Figure 3:**
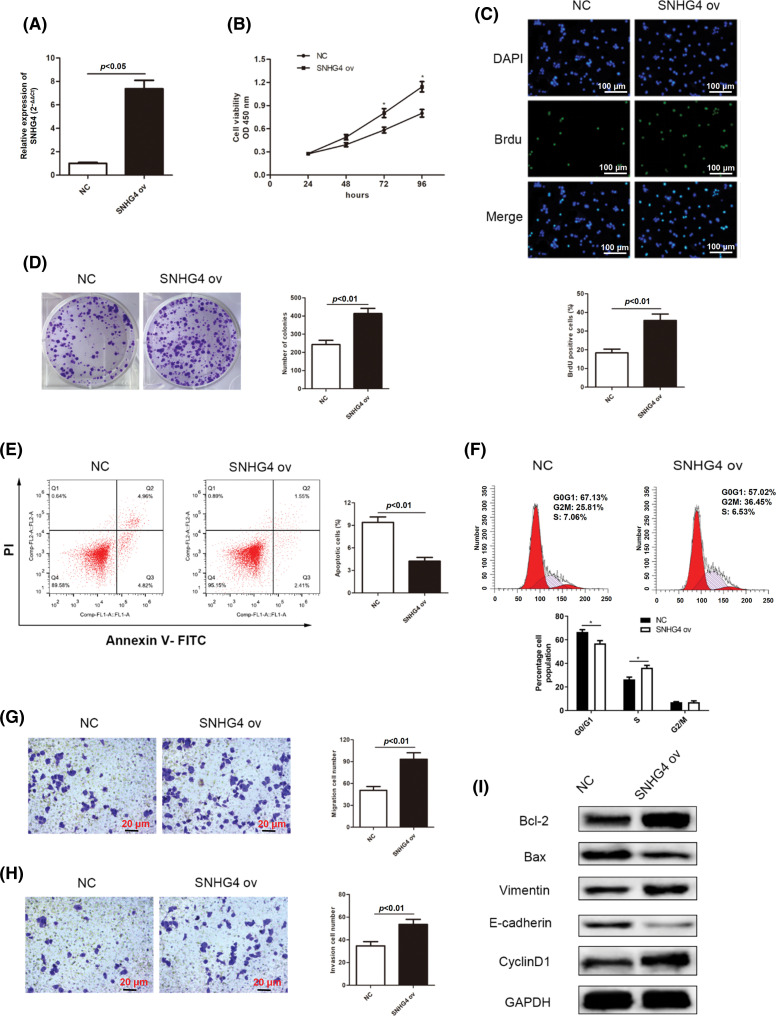
SNHG4 overexpression accelerates cell growth and metastasis in GC. (A) Establishing SNHG4 overexpression in AGS cell line. (B–D) Growth curves based on CCK-8, BrdU as well as colony formation assays of AGS cell line with SNHG4 ov transfection. (E) Cell apoptotic rate and (F) Cell cycle distribution were evaluated by flow cytometry. (G and H) Transwell assays detected invasion and migration ability of GC cells with SNHG4 overexpression. (I) Western blot assay measured expression of proteins related to proliferation, EMT and apoptosis pathways. *p < 0.05.

At the same time, the effects of overexpressed SNHG4 on the malignant behaviors of GC cells were assessed. Results from qRT-PCR assay confirmed that overexpression of SNHG4 up-regulated expression of SNHG4 in AGS cells (Fig. 3A). GC cell proliferation ability was elevated by overexpression of SNHG4 (Figs. 3B–3D). Flow cytometry analysis revealed that overexpression of SNHG4 inhibited GC cell apoptosis and declined cell cycle distribution in the G0/G1 phase (Figs. 3E and 3F). In addition, elevated SNHG4 expression promoted GC cell migration and invasion (Figs. 3G and 3H), as well as increased the protein levels of Vimentin, Cyclin D1, Bcl-2 and declined those of Bax and E-cadherin (Fig. 3I).

### SNHG4 promoted GC tumor growth *in vivo*

**Figure 4:**
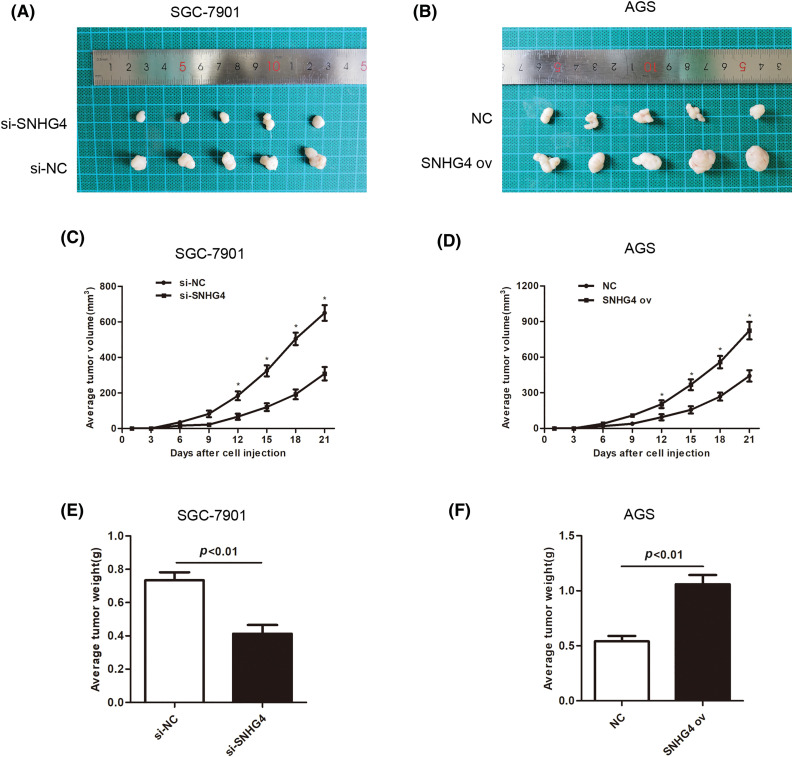
SNHG4 accelerated GC tumorigenesis. (A and B) Representative records of the size of mouse tumors derived from the corresponding GC cells SGC-7901 and AGS, respectively. (C and E) Average tumor weight and volume of SNHG4 knockdown group by injecting si-SNHG4 transfected cells (D and F) Average tumor weight and volume of the SNHG4 overexpression group by injecting SNHG4 ov transfected cells. *p < 0.05.

To fully verify the impact of SNHG4 on tumor growth, tumor xenograft of animal experiments were simultaneously performed. After 3-week adoption, SGC-7901 cells injected with si-SNHG4 indicated a reduction in tumor volume while AGS cells injected with SNHG4 ov showed an increase in tumor volume (Figs. 4A–4D). Moreover, SGC-7901 cells injected with si-SNHG4 indicated a reduction in tumor weight while AGS cells injected with SNHG4 ov showed an increase in tumor weight (Figs. 4E and 4F).

### SNHG4 bound to miR-409-3p in GC cells

**Figure 5:**
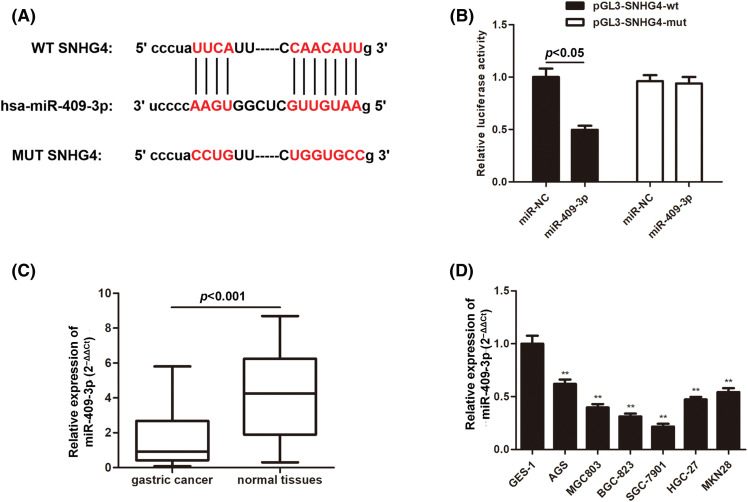
MiR-409-3p targeted SNHG4 in GC cells. (A) Bioinformatics prediction of possible binding sites of miR-409-3p with SNHG4. (B) The interaction of miR-409-3p with SNHG4 was analyzed using dual luciferase reporter assays. (C and D) Relative miR-409-3p expression in GC samples (n = 60) or normal gastric samples (n = 60) from GC patients and GC cells or GES-1 cells were subject to qRT-PCR. **p < 0.01.

Next, we set out to explore the mechanism by which SNHG4 exerted its function on GC cell progression. The bioinformatics analysis tool starBase (https://starbase.sysu.edu.cn/) confirmed that SNHG4 and miR-409-3p had possible binding sites (Fig. 5A). Moreover, the luciferase activity of pmirGLO-SNHG4-wt exhibited significant reduction by miR-409-3p overexpression, which supported the binding of SNHG4 and miR-409-3p (Fig. 5B). Additionally, miR-409-3p expression was lower in patient tumor samples (n = 60) and GC cells (Figs. 5C and 5D).

### SNHG4 promoted GC cell malignant progression by modulating miR-409-3p

**Figure 6:**
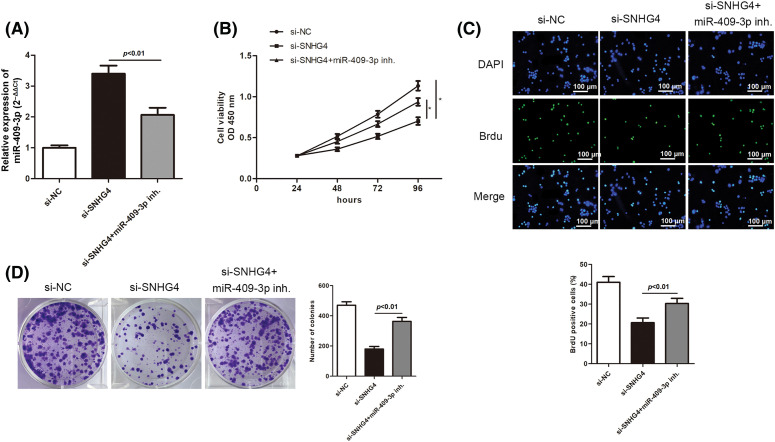

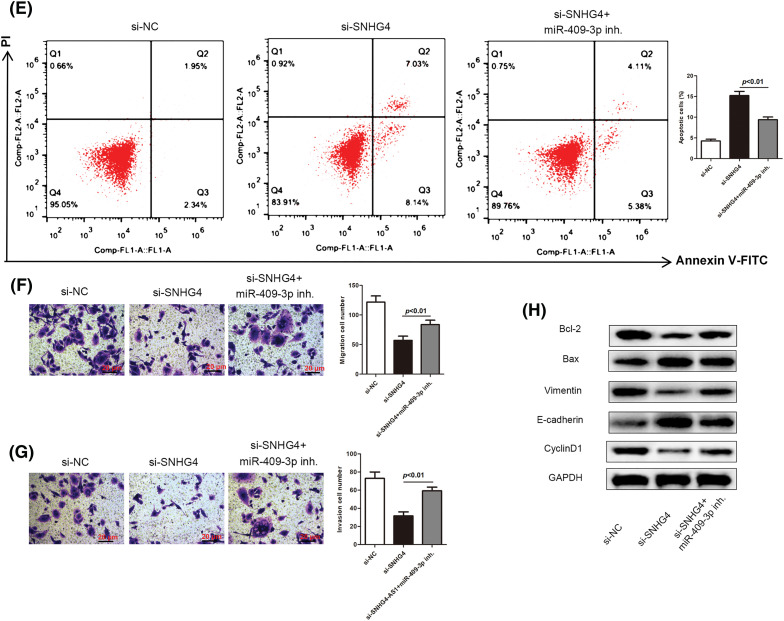
SNHG4 modulated GC cell malignancy by targeting miR-409-3p. (A) MiR-409-3p level in GC cells after transfection in the SGC-7901 cell line. (B–D) Cell proliferative capacity was evaluated with CCK-8, BrdU as well as colony formation assays. (E) Cell apoptotic rate was impaired by addition of miR-409-3p inhibitor, and evaluated using flow cytometry. (F and G) Cell migrating ability as well as invasiveness was detected transwell assays. (H) Western blot measured protein expression related to proliferation, EMT and apoptosis pathways. *p < 0.05.

We then delved into the miR-409-3p-specific biological functions in GC. SGC-7901 cells were transfected with si-SNHG4 in order to establish a SNHG4 knockdown group. Next, the si-SNHG4 and miR-409-3p inhibitor were simultaneously transfected into SGC-7901 cells to elucidate whether SNHG4 regulated GC progression through interacting with miR-409-3p. It was discovered that miR-409-3p expression was apparently elevated after SNHG4 silence, while co-transfection of miR-409-3p inhibitor offset this effect (Fig. 6A). Similar to previous findings, SNHG4 deficiency inhibited GC cells proliferation, while co-transfection of miR-409-3p inhibitor impaired this inhibition (Figs. 6B–6D). MiR-409-3p deficiency counteracted the enhanced cell apoptosis ability induced by SNHG4 knockdown (Fig. 6E). Furthermore, SNHG4 knockdown lessened the migratory and invasive abilities of GC cells, but this impact was reversed by co-transfection of miR-409-3p inhibitor (Figs. 6F–6G). In addition, the elevated protein levels of E-cadherin and Bax as well as the reduced levels of Bcl-2, Vimentin and Cyclin D1 caused by SNHG4 silence could be counteracted after synchronous miR-409-3p deficiency (Fig. 6H).

### MiR-409-3p bound to CREB1 in GC cells

**Figure 7:**
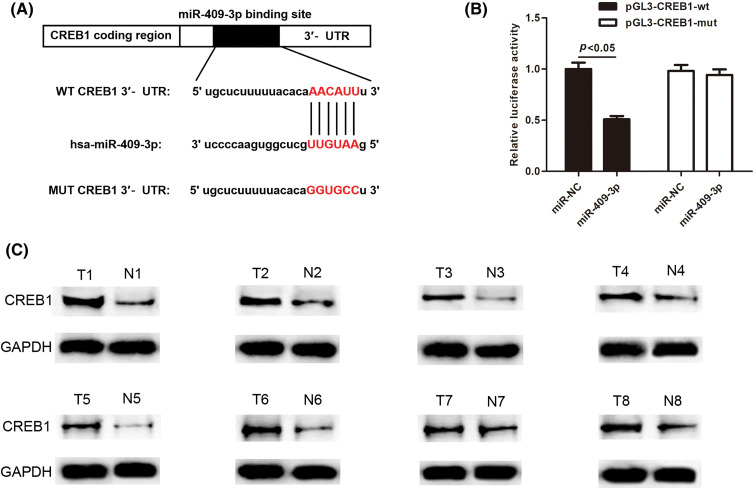
CREB1 was found to bind directly to miR-409-3p. (A) The binding sequences of CREB1 with miR-409-3p. (B) Dual luciferase assays showed the direct binding of CREB1 with miR-409-3p in GC cells. (C) Western blot detected CREB1 levels across eight pairs of GC tissues.

MiRNAs usually exert functions by modulating their target genes. Therefore, the downstream target genes of miR-409-3p were further explored. Through starBase, DIANA (https://dianalab.e-ce.uth.gr/html/diana/web/index.php?r=lncbasev2) and TargetScan (https://www.targetscan.org/vert_80/) databases, it was revealed that CREB1 had common binding sequences with miR-409-3p (Fig. 7A). To verify whether CREB1 is the target gene of miR-409-3p, dual luciferase reporter assays were conducted. The results validated that miR-409-3p overexpression sharply reduced the luciferase activity of the wild-type CREB1, but has no effect on that of mutant CREB1 (Fig. 7B). Western blot analysis revealed that CREB1 was highly expressed in eight pairs of GC patient tissues (Fig. 7C).

### Enhanced CREB1 levels rescued the suppression on GC cell malignancy affected by miR-409-3p overexpression

**Figure 8:**
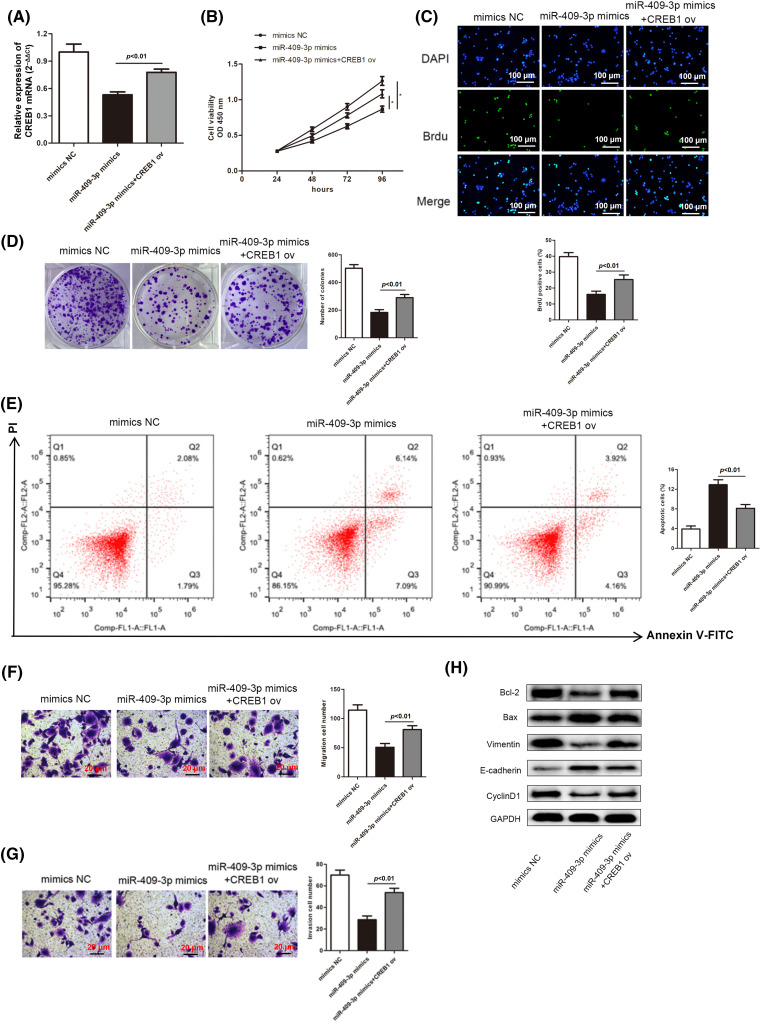
MiR-409-3p suppressed the malignant behaviors of GC cells by regulating the CREB1 levels. (A) Three groups were established by transfecting miR-409-3p mimics, miR-409-3p mimics+CREB1 ov and mimics NC in the SGC-7901 cell line. (B–D) GC cell growth was examined using CCK-8 assay kit, BrdU and colony formation experiments. (E) The apoptotic rates were subject to flow cytometry analysis. (F and G) Transwell assays determined the cell migration and invasiveness in each group. (H) Levels of proteins related to proliferation, EMT as well as apoptosis pathways were analyzed with western blot. *p < 0.05.

Next, we delved into whether CREB1 was implicated in the miR-409-3p-induced suppression on GC cell progression. Hence, the miR-409-3p mimics as well as CREB1 ov were co-transfected to SGC-7901 cells. CREB1 mRNA expression was inhibited in SGC-7901 cells transfected with miR-409-3p mimics, but was elevated after the co-transfection of CREB1 ov (Fig. 8A). Based on the functional experiments, increasing CREB1 levels were able to reverse the inhibition of miR-409-3p overexpression on GC cell proliferative capacity (Figs. 8B–8D). Consistent with this conclusion, high apoptosis rate induced by miR-409-3p elevation was impaired by simultaneous CREB1 up-regulation (Fig. 8E). Similarly, we also revealed that excessive CREB1 offset the inhibition of miR-409-3p up-regulation on GC cell migration and invasion (Figs. 8F and 8G). Moreover, the elevated protein levels of E-cadherin and Bax as well as the lessened levels of Bcl-2, Vimentin and Cyclin D1 caused by miR-409-3p increase could be counteracted after synchronous CREB1 overexpression (Fig. 8H).

## Discussion

GC is difficult to diagnose at the early stage, and most patients being diagnosed at an advanced stage, when treatment is not effective. Thus, exploring the molecular mechanisms underlying GC development may help discover potential therapeutic targets.

Substantial literatures have identified the close connection of lncRNAs with GC [29,30]. EMT is linked with high invasiveness and metastasis of tumor cells [31]. More and more studies have shown that lncRNAs function as oncogenes in GC by regulating EMT process. For example, SNHG11 is an onco-lncRNA in GC by promoting cell migration, invasion, and EMT [32]. LncRNA CHRF accelerates cell invasion and migration via EMT in GC [33].

SNHGs are a family of small nuclear RNA host genes that have been found to have abnormal functions in multiple cancer-related diseases. For example, Chen et al. detected higher expression of SNHG8 in a non-small cell lung carcinoma through interacting with miR-542-3p [34]. Meng et al. discovered that SNHG6 contributes to glioma tumorigenesis by sponging up miR-101-3p [35]. Thus far, the lncRNA SNHG4 was also investigated across several cancer diseases, including hepatocellular carcinoma [36], osteosarcoma [37], cervical cancer [38], and prostate cancer [39]. SNHG4 in GC has rarely been investigated. In this work, we initially confirmed the up-regulated SNHG4 level in GC tumor samples and cells. Functional experiments provided detailed evidence that SNHG4 enhanced GC cell malignant behaviors and EMT process. Likewise, SNHG4 accelerates colorectal cancer cell cycle and cell proliferation [40]. SNHG4 increases neuroblastoma proliferation, migration, and invasion [41]. High SNHG4 expression is an independent predictor of poor prognosis in liver cancer [42].

Previous studies have noted the importance of miRNA participants in GC. They can serve as potential biomarkers and therapeutic targets for GC [43]. Numerous studies have proven that lncRNAs interact with corresponding miRNAs, thus playing the role of ceRNA [44]. Similarly, we hypothesized that SNHG4 may interact with some certain miRNA to exert its function. Starbase database and dual luciferase reporter assays proved that miR-409-3p could bind to SNHG4. Accumulating evidence has defined the suppressor role of miR-409-3p in cancer development. For instance, miR-409-3p inhibits bladder cancer cell metastasis [45]. MiR-409-3p restrains GC cell growth and induces cell apoptosis by modulating PHF10 [46]. Consistent with the above reports, our study discovered that inhibition of miR-409-3p counteracted the suppressed GC progression caused by SNHG4 silence, which implied the tumor suppressor role of miR-409-3p in GC.

Furthermore, the target genes of miR-409-3p were also explored. Our study found that CREB1 was targeted by miR-409-3p. CREB1 has been proven to be involved in many types of tumor development [47]. CREB1 enhances the malignant behavior of liver cancer cells [48], colorectal cancer cells [49] and epithelial ovarian cancer [50]. Herei, we found that CREB1 was high-expressed in GC tissues, and CREB1 overexpression reversed the inhibition of GC cell progression mediated by miR-409-3p overexpression, reflecting the tumor promoting role of CREB1 in GC, which was consistent with previous studies [51–53].

This study has several limitations. Firstly, we did not investigate whether the SNHG4/miR-409-3p/CREB1 axis regulates GC progression via some certain signaling pathways. Moreover, further studies might be conducted to determine whether the SNHG4/miR-409-3p/CREB1 axis plays a role in GC resistance to chemotherapy reagents.

## Conclusion

Our study characterized that the lncRNA SNHG4 facilitated GC cell growth and metastasis. Further analysis revealed that SNHG4 modulated miR-409-3p/CREB1 axis to exert its function. We identified a SNHG4/miR-409-3p/CREB1 network, which may be a promising target for GC diagnosis.

## Data Availability

All data used in this research are available from the corresponding author upon reasonable request.
